# Orsay virus infection rate declines with age in *C. elegans*

**DOI:** 10.17912/micropub.biology.001434

**Published:** 2025-01-12

**Authors:** Shivaani Tishadas, Kentaro Noma

**Affiliations:** 1 Nagoya University, Nagoya, Aichi, Japan; 2 Karolinska Institutet, Stockholm, Sweden

## Abstract

The intracellular pathogen response is regulated by multiple *pals* genes in *C. elegans* . How such responses change with age is largely unknown. Thus, we investigated potential age-dependent changes in the immune response to the *C. elegans* -specific Orsay virus . When animals were exposed to equal viral concentrations, the expression of known immune-response *pals* genes and viral RNAs was lower in aged populations than in young adults. However, when young and aged populations were infected with equal viral loads, *pals* gene expression did not change with age. Therefore, aged *C. elegans* experience a decline in viral infection rate.

**Figure 1. pals gene responses to Orsay virus infection in young and aged animals:**
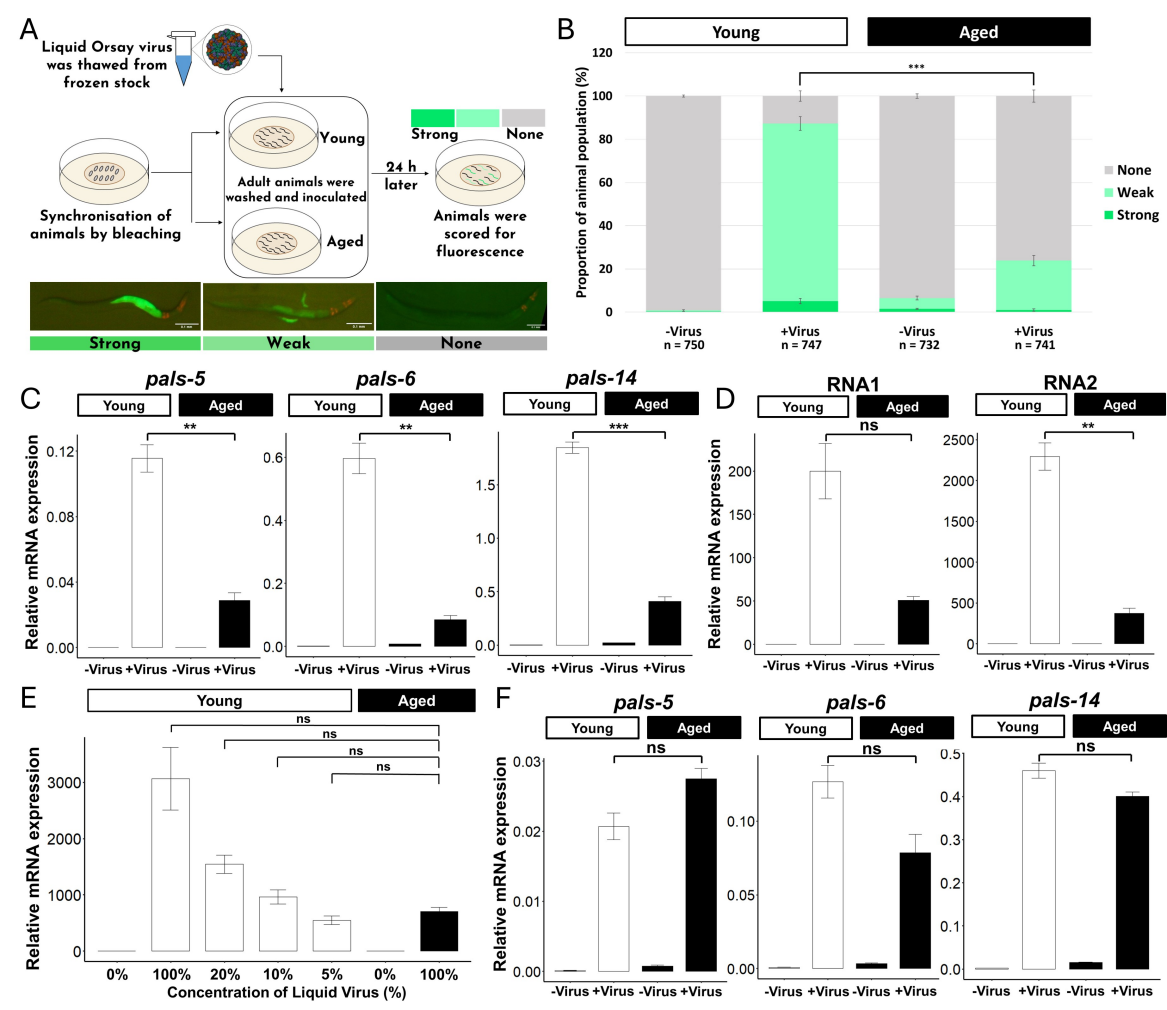
(A) Schematic of the reporter assay. ERT711 *rde-1(ne219) V; jyIs8[pals-5p::GFP + myo-2p::mCherry] X* were synchronized and inoculated with liquid Orsay virus or no-virus control at young (day 1 of adulthood) and aged (day 5 of adulthood) stages. The animals were then scored for fluorescence according to a qualitative scale. Scale: 0.1 mm. Virus image from (Tao & Guo, 2014). (B) Quantification of the reporter expression in young and aged ERT711 upon Orsay virus infection. Animals were infected with equal concentrations of the virus and scored as indicated in (A). Statistical analysis was performed using chi-squared test with continuity correction. “***”, p<0.001. (C) *pals* gene mRNA expressions in young and aged animals 24 hours post-inoculation with equal virus concentrations. mRNA expressions were quantified using qPCR in three biological replicates and compared using Student's t-test. “**”, p<0.01; “***”, p<0.001. (D) Viral RNA levels in young and aged animals 24 hours post-inoculation with equal virus concentrations. Viral RNAs were quantified using qPCR in three biological replicates and compared using Student's t-test. “ns”, p>0.05; “**”, p<0.01. (E) Viral RNA2 levels in young animals inoculated with various concentrations of liquid Orsay virus. Viral RNAs were quantified using qPCR in three biological replicates. Statistical analysis was performed by comparing the samples against aged animals inoculated with 100% liquid Orsay virus using One-way ANOVA test. “ns”, p>0.05. (F) *pals* gene mRNA expressions in young and aged animals 24 hours post-inoculation with equal viral loads. Young and aged animals were inoculated with 5% and 100% Orsay virus, respectively. mRNA expressions were quantified using qPCR in three biological replicates and compared using Student's t-test. “ns”, p>0.05.

## Description

Immunosenescence is the age-dependent decline in the immune system that potentially contributes to an increased incidence and severity of disease in elderly populations (Solana & Pawalec, 2004) . Studies in immunosenescence are vital to improving the treatment of elderly populations afflicted with disease for a better quality of life. However, causal factors for immunosenescence in mammals are poorly understood because of their complex immune systems and long-lasting ageing processes (Kim et al., 2022) . The model organism *C. elegans* is ideal for studies in ageing and immunity due to its short lifespan, genetic tractability, ease of cultivation, and simple immune system consisting of only innate responses (Brenner, 1974; Tran & Luallen, 2024) . One of these innate immune responses is the Intracellular Pathogen Response (IPR), which is regulated by genes in the *pals* family. The *pals* genes, meaning p rotein containing the ALS 2CR12 signature, regulate genes encoding ubiquitin ligase components to promote the degradation of pathogenic proteins by the ubiquitin-proteasome system (Reddy et al., 2017) . *C. elegans* uses the IPR to combat viruses; the only known naturally occurring virus specific to *C. elegans* is the Orsay virus . The Orsay virus is a positive-sense RNA virus with a bipartite genome (Guo et al., 2014) . Transmission occurs via the oral-faecal route, and infection results in an enlarged intestinal lumen and cellular structural changes (Félix et al., 2011) . Although young *C. elegans* employs immune defences, such as the IPR, against Orsay virus infection, the question of whether these responses function optimally in aged animals remains.

We used a reporter strain to easily observe changes in the animals' response to viral infection. The reporter strain ERT711 expresses a *pals-5* promoter-driven GFP localised in the intestine. More than ninety-nine percent of the adult animal population expresses GFP in the intestine when infected (Jiang et al., 2017) . For the reporter assay, animals infected with liquid Orsay virus purified from infected animals were scored for their fluorescence intensity according to a qualitative scale.

To determine the ideal inoculation time to observe maximum fluorescence, we performed a time-course experiment. Animals at day 1 of adulthood were inoculated with liquid Orsay virus and scored for fluorescence at 12-, 18- and 24-hour time points. We observed the strongest fluorescence signal at the 24-hour time point, so we set 24 hours as the viral inoculation time for further experiments.

To compare the immune responses of young and aged animals, animals at day 1 and day 5 of adulthood were inoculated with liquid Orsay virus and scored for their fluorescence intensity 24 hours later ( Fig. 1A ). Since aged animals expressed high background fluorescence, the scoring criteria were adapted to account for the high background fluorescence in animals expressing “no fluorescence”. The reporter assay revealed that aged animals had a significantly smaller proportion of fluorescent animals than the young animals ( Fig. 1B ). To define the phenotype of infection, young and aged animals were infected with liquid Orsay virus and scored for survival 24 hours later. Orsay virus has been reported not to affect the lifespan of *C. elegans* , and young animals are known to survive infection (Félix et al., 2011) . The survival assays showed that this trend persisted in aged animals. On average, young and aged animals had a survival rate exceeding 90%. We then classified the morphological phenotype of Orsay virus infection in young and aged animals. We observed that young, infected animals possessed a more disordered intestinal lumen and apical border, as well as a paler body. Infected animals were reported to have a convoluted apical intestinal border, pale bodies due to abnormal distribution of intestinal granules and reduced food consumption (Félix et al., 2011) . We observed that aged animals did not show a significant difference in body paleness when infected, but they did show convoluted intestinal structures according to varying degrees of infection.

To confirm the results of the reporter assay, we performed qRT-PCR analysis of endogenous immune response genes. In addition to the *pals-5* gene used for the reporter, *pals-6* and *pals-14* genes reported in *C. elegans* immune studies were selected (Huang et al., 2021) . For all three *pals* genes, aged animals showed lower expression levels than their young counterparts ( Fig. 1C ). To investigate if this decreased *pals* gene expression was due to a decreased viral load in aged populations, we measured the amount of viral RNA in young and aged populations. The Orsay virus has a bipartite genome made of two RNA segments, RNA1 and RNA2 (Jiang et al., 2014) . qRT-PCR analyses showed that viral RNA levels of both segments were lower in aged populations than young populations ( Fig. 1D ). This implies that the decrease in *pals* gene expression seen in aged animals can be due to a decrease in viral load. We sought to determine the concentration of liquid Orsay virus that yielded the same viral load in both young and aged animals by inoculating young animals with various concentrations of liquid Orsay virus . Young animals inoculated with 5% liquid Orsay virus showed the same viral load as that of aged animals inoculated with 100% liquid Orsay virus ( Fig. 1E ). We examined the *pals* gene expression under this condition to investigate possible changes in immune response between young and aged animals with the same viral load. Young animals inoculated with 5% liquid Orsay virus showed similar *pals* gene expression with that of aged animals inoculated with 100% liquid Orsay virus ( Fig. 1F ). Therefore, we concluded that the initial difference in *pals* gene expression between young and aged animals infected with an equal concentration of virus was likely due to a difference in the rate of viral infection and that the *C. elegans* immune response does not change between day 1 and day 5 animals.

Infection rate is defined as the infection incidence over a period of time (Reisen, 2002) ; this can be influenced by several factors, including transmission rate. The virus spreads via the oral-faecal route, so impairments in either part of said transmission route that occur with age would result in lower viral loads in the aged animals. *C. elegans* contracts a neuromuscular tube known as the pharynx to suck in food and grind them to transport to the intestine. The rate of pharyngeal contractions, or pharyngeal pumping, decreases with age (Albertson & Thompson, 1976) . Young animals have a pharyngeal pumping rate of around 300 pumps per minute, while aged animals have a rate of around 200 pumps per minute (Huang et al., 2004) . Animals also experience a reduced defecation rate with age. One study reported that animals experience a rapid decline in defecation rate from day 3 to day 6 of adulthood (Bolanowski et al., 1981) . These combined factors of reduced food intake and defecation might contribute to the reduced viral load in aged animals.

Infection rate is also dependent on viral infectivity, which is the capacity of the virus to enter the host cell and replicate by exploiting its host's resources (Rodríguez-Lázaro et al., 2013). Viral infectivity is complementary to permissivity, which is the cell's predisposition to infection (Cuccurullo et al., 2015) . Therefore, changes to cell permissivity that occur with age may adversely impact viral infectivity. A report on betanodaviruses, a close relative of the Orsay virus , described the role of the virus capsid protein encoded by its RNA2 segment in determining host specificity; mutations in the protein's primary structure greatly affected virus-host specificity and triggered a shift in receptor recognition (Low et al., 2017) . Conversely, we can hypothesize that changes in receptor expression caused by ageing may result in less efficient host cell receptor recognition. Morphological changes at the entry site could also result in less efficient viral entry. The Orsay virus is believed to enter the intestinal cells at the apical membrane (Yuan et al., 2018) . The intestinal ageing phenotype in *C. elegans* includes a more irregular and angular intestinal lumen and degradation of intestinal microvilli (McGee et al., 2011) . These changes may impact viral entry, thereby reducing viral load in aged animals. A reduced viral load can also be caused by a decrease in viral replication. Positive-sense RNA viruses such as the Orsay virus utilise the host cell's translational machinery to synthesise the products of its genome (Guo et al., 2014; Payne, 2017) . Declining rates of protein synthesis and protein turnover rates are hallmarks of ageing observed in many organisms, including nematodes (Kim & Pickering, 2023) . These could lead to a decline in viral replication, which would impact the viral load in aged animals.

Through this study, we can pose a reduced viral infection rate as a potential hallmark of early ageing. This reduced infection rate may arise from various factors, including age-related physiological changes in *C. elegans* that impact viral transmission, as well as reduced cell permissivity to the virus. However, we cannot yet say that immunosenescence does not occur in *C. elegans* . As ageing persists after day 5 of adulthood, we may see a decline in immune responses that suppress the protective effect of decreased rate of viral infection.

## Methods

***C. elegans* maintenance**

The *C. elegans* strain ERT711 *rde-1 ( ne219 ) V; jyIs8 [ pals-5 p::GFP + myo-2p::mCherry] X* was kindly provided by Emily Troemel (University of California San Diego) and maintained on a 55-mm plate, using Nematode Growth Media (NGM) at 23°C. *Escherichia coli* ( *E. coli* ) strain OP50 was cultured in LB broth for 16 hours and seeded onto the NGM plates (Brenner, 1974) .

**Preparing synchronised populations of young and aged animals**

Gravid adults were collected into 1.5 ml tubes from a confluent maintenance plate; the animals were treated with a bleaching solution (composed of 0.5 ml 1M NaOH and 0.5 ml household bleach) until the adults' bodies collapsed to release their eggs. After bleaching, the eggs were washed with sterilized water 3 times to remove debris, and centrifuged with a table-top centrifuge to precipitate the eggs. The collected eggs were diluted with 500 – 1000 µl of sterilised water. ~300 eggs were seeded onto the OP50 lawn of new NGM plates. To prevent starvation and remove larvae, day 1 animals were transferred to a new NGM plate after washing 3 times with NG buffer (51 mM NaCl, 1 mM CaCl _2_ , 1 mM MgSO _4_ , 25 mM K-PO _4_ ). This process was repeated every day until the animals reached the day 5 stage.

**Liquid Orsay virus preparation**

The *C. elegans* strain ERT711 infected with Orsay virus was kindly provided by Emily Troemel (University of California San Diego). The protocol for viral preparation was adapted from (Félix et al., 2011) . Plates of freshly starved infected worms were collected with 20 mM Tris/HCl (pH 7.0) and subjected to centrifugation at 5,000 g. The resulting supernatant was collected and centrifuged at 13,000 g; this step was repeated once. The supernatant was filtered through a 0.22 µm Millipore filter and aliquoted to be stored at -80°C.

**Reporter assay**

One hundred and twenty microliters of thawed liquid Orsay virus was spread onto NGM plates containing synchronised populations of young (day 1) or aged (day 5) animals. The plates were stored at 23°C and scored for fluorescence 24 hours later. One hour before scoring, the plates were kept at 4°C to immobilise the animals for easier and more accurate counting. Animals exhibiting strong fluorescence showed a bright signal in the intestine. Animals exhibiting weak fluorescence showed a dim fluorescence in the intestine. Animals that showed no signal were scored as having no fluorescence. The plates were blinded for unbiased scoring. For each biological replicate, three technical replicates for each sample condition were used for experiments.

**Quantitative RT-PCR**

Animals were collected 24 hours post-inoculation, washed for 20 minutes with M9 buffer (20 mM KH _2_ PO _4_ , 20 mM Na _2_ HPO _4_ , 8 mM NaCl, and 20 mM NH _4_ Cl), and stored at -80°C for more than 24 hours. RNA was extracted using RNAiso Plus (Takara Bio) and its concentration was measured using a spectrophotometer (DeNovix). The mRNA was diluted to 0.5 µg /10 µL with DEPC-treated H _2_ O (Life Technologies, Ambion®) and used to synthesise cDNA using 5x RT Master Mix (TOYOBO). cDNA was diluted twenty-five times for qPCR. For each of the three biological replicates, three technical replicates for each sample condition were used for experiments.

**Statistical analysis**

Graphs were generated using Microsoft Excel or RStudio (Team, 2020) . Images were generated using Microsoft PowerPoint. Statistical analyses were conducted using EZR (Kanda, 2024) .

## Reagents

**Table 1. List of primers used in this study**

**Table:** 

Primer	Gene	Sequence (5' → 3')
KN1170	*cdc-42*	CTGCTGGACAGGAAGATTACG
KN1171	*cdc-42*	CTCGGACATTCTCGAATGAAG
KN2157	*pals-5*	CATTGGAAAGCGATATTGGA
KN2158	*pals-5*	TCTCCAGGCACCTATCTTGTAG
KN2159	*pals-6*	TGGGTTCTGGATCAAGCAAAT
KN2160	*pals-6*	TGTTCTAGAGCTGCCTGTCTCTG
KN2161	*pals-14*	TCGGGAAAGCATCAATGAACTGC
KN2162	*pals-14*	TGTTGTGCCTCTCCTCTGCC
KN2163	OrV RNA1	TGGATCCAACGCCGTTAAC
KN2164	OrV RNA1	CGATTTGCAGTGGCTTGCT
KN2165	OrV RNA2	CCGGCGACAATGTGTACCA
KN2166	OrV RNA2	CCAGCCCTCCGTTGACAA
